# A Deadly Cargo: Gene Repertoire of Cytotoxic Effector Proteins in the *Camelidae*

**DOI:** 10.3390/genes12020304

**Published:** 2021-02-21

**Authors:** Ján Futas, Jan Oppelt, Pamela Anna Burger, Petr Horin

**Affiliations:** 1 CEITEC VFU, University of Veterinary Sciences Brno, 612 42 Brno, Czech Republic; jfutas@vfu.cz (J.F.); jan.oppelt@gmail.com (J.O.); 2 Department of Animal Genetics, Veterinary and Pharmaceutical University, 612 42 Brno, Czech Republic; 3 Department of Pathology and Laboratory Medicine, Division of Neuropathology, Perelman School of Medicine, University of Pennsylvania, Philadelphia, PA 19104-6100, USA; 4 Research Institute of Wildlife Ecology, Department of Interdisciplinary Life Sciences, University of Veterinary Medicine Vienna, 1160 Vienna, Austria; Pamela.Burger@vetmeduni.ac.at

**Keywords:** ungulates, camel, NK cells, cytotoxic T lymphocytes, perforin, granulysin, granzymes

## Abstract

Cytotoxic T cells and natural killer cells can kill target cells based on their expression and release of perforin, granulysin, and granzymes. Genes encoding these molecules have been only poorly annotated in camelids. Based on bioinformatic analyses of genomic resources, sequences corresponding to perforin, granulysin, and granzymes were identified in genomes of camelids and related ungulate species, and annotation of the corresponding genes was performed. A phylogenetic tree was constructed to study evolutionary relationships between the species analyzed. Re-sequencing of all genes in a panel of 10 dromedaries and 10 domestic Bactrian camels allowed analyzing their individual genetic polymorphisms. The data showed that all extant Old World camelids possess functional genes for two pore-forming proteins (PRF1, GNLY) and six granzymes (GZMA, GZMB, GZMH, GZMK, GZMM, and GZMO). All these genes were represented as single copies in the genome except the GZMH gene exhibiting interspecific differences in the number of loci. High protein sequence similarities with other camelid and ungulate species were observed for GZMK and GZMM. The protein variability in dromedaries and Bactrian camels was rather low, except for GNLY and chymotrypsin-like granzymes (GZMB, GZMH).

## 1. Introduction

The mammalian immune system is a complex of mechanisms able to recognize, control and eliminate pathogens or transformed cells. Mammals have evolved two major branches of immunity. Innate immune responses represent fast, often immediate, and non-specific mechanisms effective against a wide range of pathogens. Adaptive immune responses are characteristic of strong and antigen-specific reactions, slower onset, and often long-lasting immunological memory [1]. 

Both arms can recognize infected or transformed host cells and kill them by activating programmed cell death. Several effector cell types can kill directly target cells. Natural killer (NK) cells belonging to the innate arm and various populations of cytotoxic T lymphocytes, part of the adaptive arm of immunity, can use the same mechanisms to fulfill this task, which is a delivery of deadly proteins from their secretory granules to the target cell. 

Cytotoxic secretory granules are specialized lysosomes characterized by the storage of serine proteases, granzymes, and pore-forming proteins. Although they use principally the same mechanisms, various species may differ in their implementation. While human secretory granules contain a set of five granzymes (granzymes A, B, H, K, and M), a more diverse repertoire of granzymes was identified in the mouse secretory granules (granzymes A, B, C, D, E, F, G, K, M, and N) [2]. Similarly, humans use two pore-forming proteins, perforin [3] and granulysin [4], while mice synthesize only perforin, due to a lack of the granulysin gene in their genome [5]. 

Although the cellular and molecular mechanisms of killing target cells by cytolytic lymphocytes have not been fully clarified yet, it seems that an activation following the recognition of target cell, polarized trafficking of secretory granules, and exocytosis of pore-forming proteins and granzymes into the immune synapse are the main steps of the process [6]. The delivery of granzymes into the target cell is facilitated by perforin, which can bind to the cholesterol-rich plasma membrane and oligomerize to form pores [7]. Granzymes then may trigger apoptosis (granzyme B in mice) or athetosis (granzyme A in mice) [8], but they also can limit the virus replication and virion assembly (granzyme H in human) [9]. Besides activation of caspase-dependent cell death, they also can initiate caspase-independent pathways through mitochondrial damage and production of reactive oxygen species [10]. Granulysin is supposed to form pores in mitochondrial membranes that are, similarly to bacterial membranes, poor in cholesterol and rich in cardiolipin, which then leads to the delivery of granzymes [11]. The same mechanism of granulysin pore formation and entry of granzymes initiating microptosis, the programmed cell death of bacterial cells, is used for limiting the spread of intracellular bacteria from dying host cells [12].

The current knowledge of the mechanisms of cell-mediated cytotoxicity highlights the importance of the lysosomal cargo in T and NK cells and of genes encoding these proteins. In humans, different pathological phenotypes were associated with various mutations in the perforin gene, including e.g., a genetic disorder, the familial hemophagocytic lymphohistiocytosis, protracted viral infections, and/or susceptibility to hematological malignancies [13]. On the other hand, no mutation leading to granulysin deficiency has been described in humans so far, and there is no evidence that promoter and/or coding sequence polymorphisms would affect granulysin functions [11]. Likewise, no genetic disorder caused by a deficiency of a particular granzyme has been recognized in humans or mice, probably due to a partial overlap in the functions of different granzymes. 

Most of the data on cytotoxic effector proteins were collected on model organisms, humans, and laboratory rodents. Much less is known about these important immune mechanisms and their molecules in domestic animals. To date, there is no direct evidence of functional perforin, granulysin, or granzymes in camelids. The species with at least partly characterized cytotoxic effector proteins of secretory granules, which is the most closely related to camelids, is cattle. Expression of bovine perforin was tested in the endometrium during the estrous cycle [14]. Functional bovine homologs of granulysin were identified [15,16]. Recently, a novel granzyme O gene was identified in bovids, and expression of perforin and six bovine granzymes (A, B, H, K, M, and O) was tested in resting peripheral blood mononuclear cells and populations of activated CD4, CD8, and γδ T lymphocytes and NK cells [17]. 

Although they represent an important part of the immunogenome, genes encoding these effector molecules have so far been only poorly annotated and characterized in Old World camels as well as in other camelids, and only limited information thus can be retrieved from the currently available genomic assemblies of different camelid species.

Therefore, the objective of this work was to provide a detailed annotation of genes contributing to the functional potential of effector immune cells in Old World camels and to compare this part of the camel immunogenome to New World camelids, and in a broader context, to their closest relatives, even-toed and odd-toed ungulates. 

## 2. Materials and Methods

### 2.1. Genomic Resources

Reference genome assemblies currently available for selected species of ungulates were accessed and searched at the National Center for Biotechnology Information (NCBI) database RefSeq [18]. They included *Camelus dromedarius* (dromedary camel) assembly CamDro3 (accession code GCF_000803125.2), *Camelus bactrianus* (Bactrian camel) Ca_bactrianus_MBC_1.0 (GCF_000767855.1); *Camelus ferus* (wild two-humped camel) BCGSAC_Cfer_1.0 (GCF_009834535.1); *Vicugna pacos* (alpaca) VicPac3.1 (GCF_000164845.3); *Bos taurus* (cattle) ARS-UCD1.2 (GCF_002263795.1); *Bos indicus* (zebu cattle) Bos_indicus_1.0 (GCF_000247795.1); *Bos mutus* (wild yak) BosGru_v2.0 (GCF_000298355.1); *Capra hircus* (goat) ARS1 (GCF_001704415.1); *Ovis aries* (sheep) Oar_rambouillet_v1.0 (GCF_002742125.1); *Sus scrofa* (swine) Sscrofa11.1 (GCF_000003025.6); *Equus caballus* (horse) EquCab3.0 (GCF_002863925.1) and *Equus asinus* (donkey) ASM130575v1 (GCF_001305755.1). To analyze mRNA or protein sequences, a contig sequence containing a complement of *NK-lysin* genes of *Bos taurus* was accessed at GenBank [19] (KT715031). 

### 2.2. Animals

Two panels, each composed of ten camels of the domestic species, were selected from samples collected during previous projects (see Table S1). The samples were selected randomly from each geographic region represented in our archived collection of samples. The *C. dromedarius* panel consisted of individuals from Jordan (Irbid), Iran, Saudi Arabia (Magaheem and Wadda), Canary Islands, UAE (Dubai), Kenya, Sudan, Nigeria, and Kazakhstan. Genomic DNA samples previously isolated by an improved salting-out DNA extraction [20] and kept frozen at −20 °C originated from plucked hair or EDTA-anticoagulated blood collected on Whatman FTA^®^ cards (Sigma-Aldrich, Vienna, Austria) during routine veterinary controls (Austrian Science Fund (FWF) P1084-B17 and P24706-B25; PI: P. Burger). 

The *C. bactrianus* panel consisted of individuals from three Mongolian regions (Bayan Ovoo, Galshar, and Norovlin). All the Bactrian camel samples were collected during veterinary procedures for a previous research project (GACR 523/09/1972; PI: P. Horin). The genomic DNA was isolated from archived FTA cards with EDTA-anticoagulated whole blood samples by NucleoSpinBlood© Kit (Macherey-Nagel, Düren, Germany) according to the manufacturer’s protocol.

### 2.3. Phylogenetic Analysis

Current annotations of the ungulate reference genomes were searched for perforin (*PRF1*), granulysin (*GNLY*), and granzyme (GZM*) genes. The genomes contained adequately annotated genes; however, they still were cross-checked using the tblastn algorithm of NCBI’s BLAST^®^ [21] with sequences of human PRF1 (NP_001076585.1), GZMA (NP_006135.2), GZMB (NP_004122.2), GZMH (NP_219491.1), GZMK (NP_002095.1), GZMM (NP_005308.2), along with mouse GZMC (NP_034501.2), GZMD (NP_034502.2), GZME (NP_034503.2), GZMF (NP_034504.1), GZMG (NP_034505.1), GZMN (NP_694692.1), and cattle GZMO (NP_001001142.1) and GNLY (NP_001068611.1). Retrieved model mRNA and predicted protein sequences were downloaded in the FASTA format and checked for inconsistencies. Some sequences must be corrected based on cross-species alignments of mRNA to the genome by SPLIGN [22] or tblastn BLAST^®^ search of the protein sequence in whole-genome shotgun contigs coming from different animals of the same species. 

All sequences were then used for subsequent phylogenetic analyses. A full list of their accession numbers is in Table S2. Each of the two final sets of protein sequences (pore-forming proteins and granzymes) was aligned separately using the CLUSTALW algorithm in MEGA X [23] software. The alignments were analyzed for phylogenetic relationships using the Neighbor-Joining method and the evolutionary distances were computed using the p-distance method in MEGA X software. All ambiguous positions were removed for each sequence pair. The evolutionary tree was constructed and bootstrap tested by 1000 replications. The human complement factor D (CFD, accession NP_001919.2) was used as an outgroup for a set of granzymes.

### 2.4. Re-Sequencing of Genes

Two genes encoding pore-forming proteins and six granzyme coding genes were genotyped by next-generation sequencing of long-range PCR amplicons in the panels of ten dromedary and ten Bactrian camels. Gene-specific primers encompassing full-length genes were designed based on NCBI *Camelus dromedarius* gene sequences using Primer-BLAST [24] and checked for specificity against reference genomes of both camel species. The list of primer pairs used, along with corresponding annealing temperatures is in Table S3. All PCR characteristics and corresponding thermal profiles are summarized in Table S4. Briefly, the KAPA 2G Robust HotStart PCR Kit (Kapa Biosystems, Cape Town, South Africa) was used for amplicons up to 6 kilobases and Expand Long Range dNTPack (ROCHE Diagnostics, Mannheim, Germany) for longer amplicons. PCR products were checked by 0.5% agarose gel electrophoresis with Midori Green Advance DNA stain (Nippon Genetics Europe, Düren, Germany) fluorescent dye for visualization. They were quantified by Invitrogen™ Qubit™ Fluorometer using Qubit™ dsDNA BR Assay Kit (Thermo Fisher Scientific, Waltham, MA, USA) and kept frozen at −20 °C until massive parallel sequencing. Long-range PCR amplicons were indexed separately for each gene and individual camel during library preparation using the Nextera XT DNA Library Preparation Kit (Illumina, San Diego, CA, USA). The resulting libraries were sequenced on a MiSeq™ System (Illumina, San Diego, CA, USA) platform using the MiSeq™ Reagent Kit v2 (500-cycles) according to the manufacturer’s protocol in two separate runs. The quality of the raw sequencing reads was checked using FastQC version 0.11.9 [25]. Low-quality read ends were removed by Trimmomatic version 0.39 [26] (SLIDINGWINDOW:4:15). Only reads longer than 150 nucleotides were used for mapping by BWA-MEM version 0.7.15 [27] to *C. dromedarius* adequate reference sequence for each amplicon. The alignment was post-processed using SAMtools version 1.4.1 [28] (sorting and conversions), GATK version 3.5 [29] (indel realignment), and Picard version 2.20.4 [30] (PCR duplicates removal). Only mappings with a minimal mapped length of 70 nucleotides, a maximum of 5% soft-clipping, and a maximum of 10% mismatches were kept using NGSUtils version 0.5.9 [31] and BBMap version 38.58 [32]. The average coverage per amplicon varied approx. from 40X to 4000X, with a median of 908.5478X. The statistics for individual sequences are listed in Table S5. Alignments of reads to the reference sequence were inspected using the IGV software version 2.8.13 [33]. Variable positions detected in the homozygous condition, along with insertions/deletions were assigned to consensus sequences for each animal. Confirmed sequence variants detected in the heterozygous condition were assigned to consensus sequences using the IUPAC nucleotide ambiguity codes in BioEdit version 7.2.6 [34]. 

### 2.5. Analysis of Genetic Polymorphisms

Sequences obtained from camels of the same species by re-sequencing were manually aligned, separately for each gene. Numbers of single nucleotide polymorphisms (SNPs) were counted using DnaSP version 6.12.03 [35]. cDNA sequences were inferred in silico using BioEdit v.7.2.6, based on C. dromedarius mRNA models for each gene (see Table 1) and checked by SPLIGN for completeness. cDNA haplotypes of each individual in the species panel were reconstructed for each gene separately using the PHASE [36] algorithm and numbers of SNPs were counted in DnaSP v.6.12.03. Coding sequences were extracted and amino acid sequences were deduced by in silico translation using BioEdit v.7.2.6. Sequences differing in at least one amino acid position were numbered and designated as different haplotypes of a particular gene. The predicted protein variants for both camel species were aligned with C. ferus and V. pacos reference sequences by CLUSTALW and compared. 

## 3. Results

**Figure 1:**
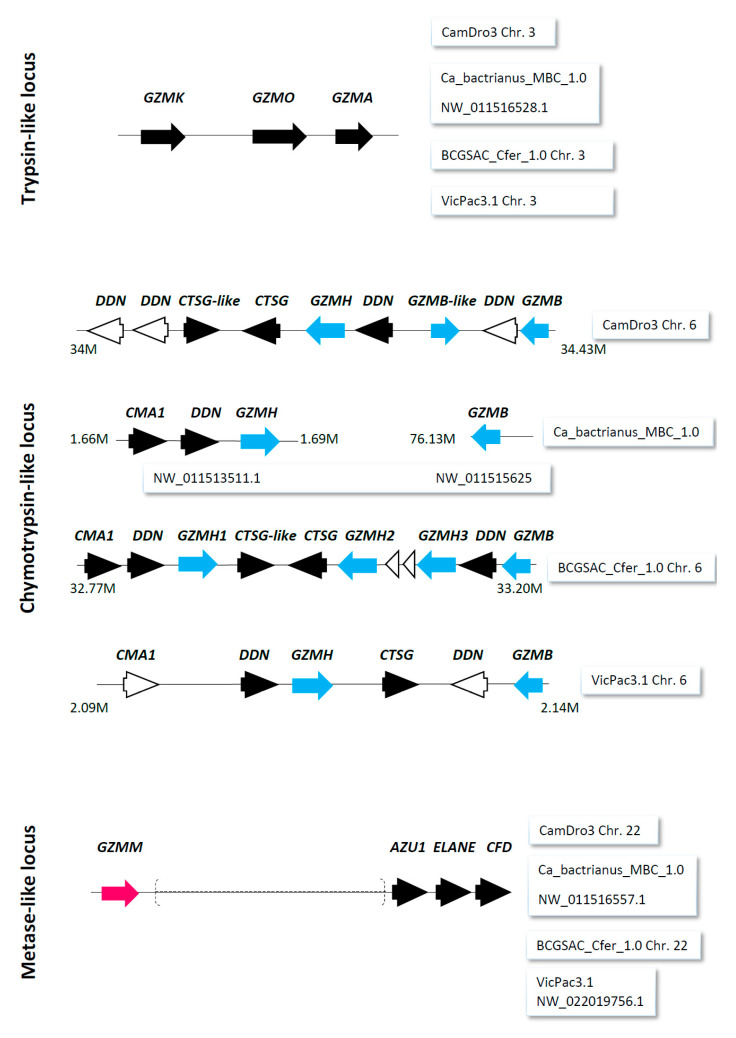
Genomic organization of granzyme genes in camelids. Three separate genomic regions (trypsin-like, chymotrypsin-like, and metase-like locus) contain granzyme coding genes (*GZMA*, *GZMB*, *GZMH*, *GZMK*, *GZMM*, and *GZMO*) in NCBI reference genomes of camels and alpaca (see tags). Other closely related potentially functional genes (full arrows) or pseudogenes (empty arrows) were duodenase (*DDN*), cathepsin G (*CTSG*), chymase (*CMA1*), azurocidin (*AZU1*), neutrophil elastase (*ELANE*), and complement factor D (*CFD*). The presence of non-homologous genes in the metase-like locus is indicated by parentheses. Coordinates are given in megabases (M). cyan, magenta—highlight granzyme genes.

For camelids, a survey of the reference genomes revealed potentially functional single-copy genes coding for pore-forming proteins, perforin, and granulysin. A set of genes encoding granzymes distributed in three chromosomal regions was identified. Genes encoding the trypsin-like and metase-like granzymes were annotated as single-copy genes in all searched assemblies, while for genes coding for chymotrypsin-like granzymes B and H (*GZMB* and *GZMH*, respectively), variation in the number of loci between assemblies were observed (Figure 1). In the domestic Bactrian camel and alpaca genomes single copy *GZMB* and *GZMH* genes were found; the dromedary camel reference genome contained two copies of *GZMB* and one copy of *GZMH*, while in the wild camel genome, one copy of *GZMB* and three copies of *GZMH* were found. 

Different numbers of genes/copies were also observed in other ungulates genome assemblies. In all searched ungulate genomes, the perforin gene was also present as a single copy gene. In contrast to camelid genomes, the number of granulysin genes varied from a single copy in most assemblies to two copies in goat and sheep genomes. All bovine reference genomes possessed only one copy of *GNLY*/*NK-lysin*, although a copy number variation up to four genes was previously described in cattle [16]. 

Similar to camelid genomes, the most variable number of copies was observed for the chymotrypsin-like-locus with *GZMB* and *GZMH* genes. Single genes were identified in swine, wild yak, zebu cattle, while single *GZMB* and two *GZMH* were present in cattle, accompanied by some pseudogene sequences. Likewise, two functional copies of *GZMB* and a single *GZMH* were found in the goat genome and two full copies of both genes were present in the sheep reference genome, along with some fragmentary unidentified sequences. The horse genome contained ten copies of *GZMB* and fourteen of *GZMH*, while the donkey genome possessed twelve *GZMB* and eight *GZMH* loci, accompanied with different numbers of pseudogenes/gene fragments in both species. The trypsin-like and metase-like loci contained single-copy genes *GZMK*, *GZMO*, *GZMA*, and *GZMM* in all ungulates. All granzyme genes were potentially functional, except the pseudogenized *GZMO* in the horse and donkey reference genomes.

### 3.1. Phylogenetic Analysis

**Figure 2:**
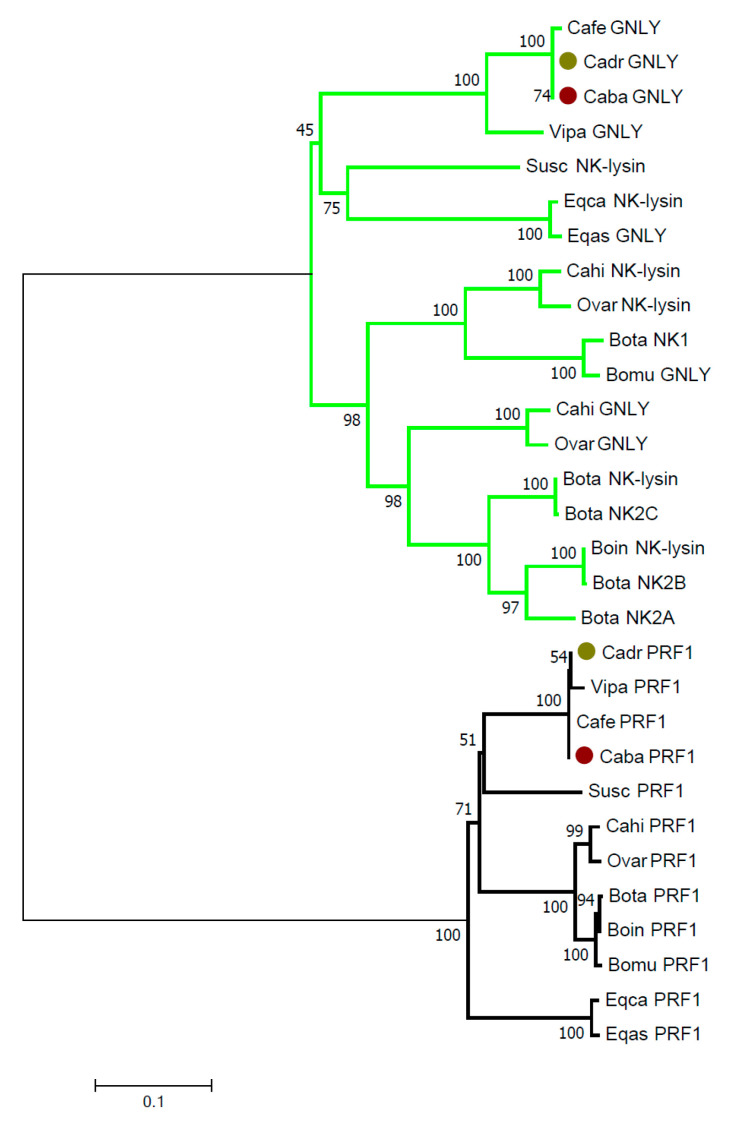
Phylogeny of pore-forming proteins in domesticated ungulates. Amino acid sequences for perforin (PRF1) and granulysin (GNLY)/ NK-lysin of dromedary (Cadr) and Bactrian camel (Caba), highlighted by color dots, were compared to reference sequences of wild camel (Cafe), alpaca (Vipa), swine (Susc), goat (Cahi), sheep (Ovar), cattle (Bota), zebu cattle (Boin), wild yak (Bomu), horse (Eqca), and donkey (Eqas) (for accession numbers see Table S2 and KT715031 for bovine NK-lysins NK1, NK2A, NK2B, and NK2C), and analyzed in MEGA X. The evolutionary history was inferred by the Neighbor-Joining method and the evolutionary distances were computed using the p-distance method and are given in the units of amino acid differences per site. The percentages of concordant replicates in the bootstrap test (1000 replicates) are shown next to the branches. Bold—pore-forming protein for eukaryotic membranes; green—pore-forming proteins for prokaryotic (mitochondrial) membranes.

A comparison between pore-forming perforins (eukaryotic cell membrane) and granulysins (prokaryotic cell membrane) found in ungulates is in Figure 2. It shows high homologies of PRF1 amino acid sequences within the ungulate families/sub-families *Camelidae, Bovidae* with *Caprinae, Bovinae*, and *Equidae*, but a clear separation of these families. The swine PRF1 sequence is homologous to camelid sequences and is located in the tree as their closest relative. Amino acid sequence similarities of GNLY/NK-lysin are high only in camels and equids. These sequences are closest to the alpaca and swine sequences, respectively. The ruminant sequences are diversified according to the locus. Four bovine loci formed two groups of related sequences.

**Figure 3:**
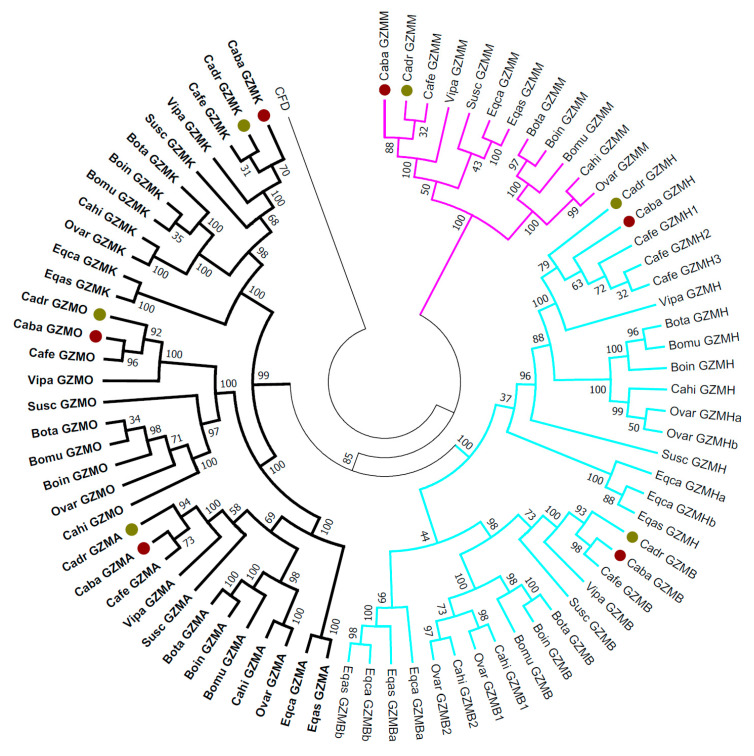
Phylogeny of granzymes in domesticated ungulates. Amino acid sequences for granzyme A (GZMA), granzyme B (GZMB), granzyme H (GZMH), granzyme K (GZMK), granzyme M (GZMM), and granzyme O (GZMO) of dromedary (Cadr) and Bactrian camel (Caba), highlighted by color dots, were compared to reference sequences (for accession numbers see Table S2) of wild camel (Cafe), alpaca (Vipa), swine (Susc), goat (Cahi), sheep (Ovar), cattle (Bota), zebu cattle (Boin), wild yak (Bomu), horse (Eqca) and donkey (Eqas), and analyzed in MEGA X. The evolutionary history was inferred by the Neighbor-Joining method and the evolutionary distances were computed using the p-distance method. The bootstrap consensus tree rooted to human complement factor D (CFD) is presented and the percentages of concordant replicates in the bootstrap test (1000 replicates) are shown next to the branches. Bold—trypsin-like locus; cyan—chymotrypsin-like locus; magenta—metase-like locus.

An overview of phylogenetic relationships among six types of granzymes in ungulates is in Figure 3. The granzyme amino acid sequences formed three main clades according to the chromosomal region and supposed specificity (trypsin-like, chymotrypsin-like, and metase-like). Within each granzyme sub-clade, separation of odd-toed ungulates (*Equidae*) and even-toed ungulates (*Bovidae, Suidae,* and *Camelidae*) was observed, except for GZMO (not present in equids) and GZMM, whose swine and equid sequences are highly homologous. The camelid sequences grouped mostly with swine sequences as sister clades, except GZMO and GZMH.

### 3.2. Analysis of Genetic Polymorphisms

The results of genotyping the panels of dromedary and Bactrian camels in cytotoxic effector protein-coding genes by resequencing long-range PCR amplicons are summarized in Table 1. Two dromedary camels were identified to be probably hybrids of mixed ancestry (*C. dromedarius* × *C. bactrianus*); therefore, their genotypes were excluded from the final set of sequences analyzed. An example of such an individual (# 891) is in Figure S6; a comparison with Figure S7 documents the presence of a *C. bactrianus* haplotype. The variability of the dromedary gene sequences reached low values for all but two genes (*GZMH* and *GZMM*) and was lower than the variability of Bactrian gene sequences in all but two genes (*PRF1* and *GZMM*). The most variable genes were *GNLY*, *GZMH*, and *GZMB* in Bactrian camels. They also coded for the most variable protein sequences, with four to five variants per gene. The majority of genes revealed basic levels of protein variability, with one or two protein variants. The comparisons of identified protein variants with sequences from wild camel and alpaca can be found in the Supplementary Materials.

The perforin protein identified in Bactrian camels was identical to the reference sequence of the wild camel (Figure S1). The alpaca PRF1 differed in five amino acid (AA) positions from camel proteins.

The granulysin protein of dromedary camels was identical to one of the GNLY variants found in Bactrian camels (Figure S2). Another Bactrian camel variant was shared with wild camels. The alpaca GNLY differed from the camel sequence in sixteen AA positions.

Granzymes encoded by the trypsin-like locus showed minimal polymorphism in both dromedary and Bactrian camels (Figure S3). The granzyme K was invariant in all camels and differed by only one AA from GZMK of alpaca. One of the granzyme O variants of Bactrian camels was identical to the wild camel reference sequence, the second differed by only one AA. The alpaca GZMO differed in eight to nine AA positions from the GZMO of camels. The granzyme A variants of Bactrian camels differed by one AA in the presumed S1 pocket, determining the enzyme’s substrate specificity. The second variant was identical to the wild camel reference sequence. The alpaca GZMA differed altogether in ten to eleven AA positions from proteins of camels and AA change in the S3 pocket distinguished alpaca from all camel sequences.

The chymotrypsin-like locus granzymes were the most polymorphic proteins in dromedary and Bactrian camels (Figure S4). One of the granzyme B variants identified in Bactrian camels was identical to the wild camel GZMB sequence. Only one Bactrian camel GZMB differed within the substrate determining pockets S1 and S3 of the enzyme molecule. The alpaca GZMB sequence differed by thirteen to nineteen AA positions from the dromedary and Bactrian camel variants. Comparably to GNLY, one of the granzyme H variants of Bactrian camels was shared with dromedaries, while another one was identical to the wild camel GZMHs. The alpaca GZMH was distinguished by five AA species-specific positions and three AA positions were shared with variable positions in camels.

The metase-like locus granzyme M was the least polymorphic protein in both dromedary and Bactrian camels (Figure S5). This invariant camel GZMM differed by two AA positions from alpaca GZMM, first in the signal peptide and second in the S3 pocket of the mature enzyme.

## 4. Discussion

Cytotoxic T cells and NK cells can kill various target cells based on their expression and release of toxins, including the pore-forming protein perforin, granulysin, and serine protease granzymes. As such, they are important players in immune responses and the underlying genes are subject to strong evolutionary pressures exerted by pathogens. Although camelids represent an important model for comparative immunological and immunogenetic studies [37], we have virtually no knowledge of this part of their immune system and immunogenome. This study aimed to address this issue by using comparative genomic approaches. Considering the scarcity of data, we analyzed not only the Old World camels, but also a representative of New World camelids (alpaca), and their closest relatives, even-toed and odd-toed ungulates, for which informative genomic data were available. 

The major limitation of this comparison is the fact that the currently available reference genomes were obtained by various massive parallel sequencing techniques, mostly by short-read sequencing. This approach does not allow to fully resolve complex organization of some genomic regions, containing repetitions and gene duplication. Highly similar loci and identical loci cannot be distinguished, and their numbers thus remain unrevealed by the process of annotation. 

This is also an obstacle for analyzing inter- and intra-specific genetic variability. It is not always clear whether observed inter-specific differences in the numbers of loci and their sequences are real or whether they are artifacts due to technical limitations of sequencing and bioinformatic procedures [38]. For example, the *GZMB-like* gene (LOC116153371) annotated in the CamDro3 assembly is not fully covered by RNA-seq data. Despite its 96% homology to the nucleotide sequence of our *GZMB* amplicon, the identity of the predicted GZMB-like protein (XP_031308642.1) to the dromedary GZMB protein is only 42%. The catalytic triad of such a GZMB-like protein is then mutated. Without a careful re-annotation, results of phylogenetic and selection analyses of functionally important multiplicated loci thus may be biased. “*In silico*” proteomic analysis could be an approach to validate genomic data. However, no hits for camelids were identified in the Swissprot and PDB databases, so no validation could be made. In general, data validation of this type is often impossible in non-model mammalian species due to a complete lack of information.

Duplications and copy number variation can also be observed within species. In the context of this work, granulysin loci of bovids represent an informative example. The reference genomes of bovids tested here possessed one copy of the granulysin coding gene. However, a copy number variation with up to four genes per individual was reported in cattle [16]. This probably led to confused annotations of granulysin loci in the entire family *Bovidae*, which got different names in cattle, zebu cattle, and wild yak compared to sheep and goat. In this study, we were confronted with the same issue for the granzyme H locus *GZMH* in the *Camelidae*. The majority of genomes (dromedary, Bactrian camel, and alpaca) contain a single annotated gene, while the recently published wild camel reference genome harbors three copies of *GZMH* [39]. This assembly is based on long-read sequencing, while the domestic Bactrian assembly is based on short-read sequencing, and the dromedary and alpaca assemblies are hybrid assemblies produced by combining short-read sequencing, chromosome conformation capture, and long-read sequencing [40,41]. The technical issue is that domestic Bactrian camels and dromedaries may also possess more *GZMH* copies. However, as we were unable to design locus-specific primers, our primers can amplify all three loci of the wild camel. 

On the other hand, we were able to resolve inter-specific differences in the analyzed group of ungulates. In the species analyzed, we found no gap in the set of genes determining the cargo of cytotoxic granules of effector immune cells. All reference genomes possessed at least one copy of perforin and granulysin coding genes, as well as of each of six cytotoxic granule serine proteases, the granzymes. The only exception is the granzyme O in equids, which miss a functional GZMO protein due to the presence of a pseudogene in this locus. On the other hand, a rather extensive variation in the number of loci was observed for some of the genes analyzed, namely genes coding for granulysin in the family *Bovidae* and granzymes B and H in the families *Bovidae* and *Equidae*. The most complex genomic organization was found in the horse (EquCab3.0—chromosome 1 and contigs 4933, 7538, 26253, and 39589) and donkey (ASM130575v1—scaffolds 170, 647, and 1131) genomic regions containing many copies of *GZMB* and *GZMH* genes and pseudogenes.

Despite the limitations mentioned above, we are confident that our phylogenetic analyses reflect well evolutionary relationships between the proteins analyzed. We focused on protein rather than nucleotide sequences. Proteins are under direct selective pressures, which allow a better evaluation of effects shaping the underlying genes. The phylogenetic trees constructed for perforin and granulysin (Figure 2) revealed different evolutionary pathways of these two pore-forming proteins. The perforin sequences remained highly similar in all ungulates, reflecting however the evolutionary history of the group, with no deviation from the standard zoological taxonomy. It thus seems that purifying selection was the major evolutionary force shaping the gene product as a whole. On the other hand, granulysin amino acid sequences were more diversified and showed some deviations from the standard zoological taxonomy, unexpectedly placing equid sequences as a sister clade to the swine sequence. Analysis of positive selection on all the sequences, which is an independent task beyond the scope of this study, should provide a more detailed insight into selective forces acting on specific amino acid positions.

The tree-based on granzymes (Figure 3) matched well the standard zoological tree of ungulates. Slight discrepancies were observed mainly for swine sequences, which were placed next to equid sequences for granzymes H and M. The tree also reflects the missing functional protein of the granzyme O in the horse and donkey.

The genetic polymorphisms of genes and their protein products were tested in the panels of dromedary and domestic Bactrian camels. Retrieved on low numbers of camels, the data cannot be interpreted in terms of population diversity; however, they provide primary information about polymorphic patterns of the genes analyzed. While all eight genes could be re-sequenced in all domestic Bactrian samples, small amounts of genomic DNA from some dromedaries proved eventually to be insufficient for all necessary analyses and had to be replaced during the study. Therefore, the final data on eight genes were not always retrieved from the same set of 10 individuals. Based on sequencing, two dromedaries were identified as heterozygotes for dromedary and Bactrian camel sequences in some genes. These sequences were excluded from the variability analysis and reconstruction of haplotypes. The camelid variants of the perforin protein were compared with a list of known human missense mutations [13], but no AA position matched. The possible functional importance of the perforin variants observed in dromedary camels remains to be studied, similarly to granulysin variants found in Bactrian camels. Out of four possible primary protein variants, only three forms of the predicted mature protein differing in two AA positions were identified. Functional analysis and a comparison of alpaca and camel granulysins would be interesting as they differ in ten amino acids. 

A spectrum of six granzymes could be predicted to exist in cytotoxic granules of camelids. All six camelid proteins have key features of serine proteases, with the catalytic triad and the substrate-determining pockets S1, S2, and S3. As the camelid granzymes K, O, and A encoded in the trypsin-like locus have the same set of AA residues in primary substrate-determining pocket S1 as their bovine counterparts, they are likely to have a tryptase activity evidenced in other mammalian species [17]. The camelid granzymes B and H encoded in the chymotrypsin-like locus showed more divergence in the secondary substrate-binding pockets S2 and S3 than their bovine counterparts, while motifs of the S1 pocket remained conserved. The camelid granzymes B and H are thus likely to have aspase and chymase activity, respectively. The activity of the particular Bactrian GZMB variant with glutamine in S1 is hard to predict, as for the mouse GZMC, its most comparable mammalian counterpart, no known activity was identified. Due to a substitution of proline present in the S1 pocket of the human, mouse, and bovine enzymes by serine in camelids, it is difficult to predict the metase activity of the camelid granzyme M. No protein variant identified showed possible deleterious amino acid change.

Expression analysis of granzyme genes in various cytotoxic cells and testing substrate specificity of variant proteins, which would help to address these issues is hampered by the lack of specific antibodies to sort populations of immune cells in camels [42]. At this stage, our data provide more information about the immunogenome in camels, representing in many regards a special immunological model [43]. The data on genetic polymorphisms may contribute to a better understanding of an apparently unusually low diversity of camelid genomes with a special focus on the position of immunity genes [44]. SNP loci and haplotypes identified in the genes analyzed may also serve as helpful markers in various types of association studies.

## 5. Conclusions

The data showed that all extant Old World camelids possess functional genes for two pore-forming proteins (PRF1, GNLY) and six granzymes (GZMA, GZMB, GZMH, GZMK, GZMM, and GZMO). All these genes were represented as single copies in the genome except the GZMH gene exhibiting interspecific differences in the number of loci. High protein sequence similarities with other camelid and ungulate species were observed for GZMK and GZMM. The protein variability in dromedaries and Bactrian camels was rather low, except for GNLY and chymotrypsin-like granzymes (GZMB, GZMH).

## Figures and Tables

**Table 1:** Genetic polymorphisms of cytotoxic granule proteins in dromedary and Bactrian camels.

Locus	Species	No. of Animals	Amplicon Length (bp)	No. of SNPs	mRNA Reference	mRNA Length (nt)	No. of SNPs	No. of Haplotypes	No. of Proteins
*PRF1*	*C. dromedarius*	9	4638	4	XM_031461138	2414	3	4	2
	*C. bactrianus*	10	4637	2		2414	2	3	1
*GNLY*	*C. dromedarius*	9	4341	7	XM_010997841	700	0	1	1
	*C. bactrianus*	10	4352	59		700	7	6	4
*GZMK*	*C. dromedarius*	10	9416	5	XM_010977773	1126	1	2	1
	*C. bactrianus*	10	9416	13		1126	2	2	1
*GZMO*	*C. dromedarius*	9	10,201	4	XM_031440963	988	0	1	1
	*C. bactrianus*	10	10,213	19		998	2	2	2
*GZMA*	*C. dromedarius*	9	7612	3	XM_010977771	1039	0	1	1
	*C. bactrianus*	10	7612	8		1039	3	4	2
*GZMB*	*C. dromedarius*	9	3421	4	XM_010986180	987	1	2	3
	*C. bactrianus*	10	3421	25		986	9	5	5
*GZMH*	*C. dromedarius*	10	5563	14	XM_031453514	950	4	4	2
	*C. bactrianus*	10	5561	49		950	8	8	4
*GZMM*	*C. dromedarius*	10	5908	12	XM_010985277	943	0	1	1
	*C. bactrianus*	10	5914	4		943	0	1	1

Bp—basepairs, nt—nucleotides, SNP—single nucleotide polymorphism.

## Data Availability

The sequences obtained for dromedary and domestic Bactrian camels were deposited to GenBank [19] under accession numbers MW456757-MW456916.

## Supplementary Materials

The following are available online at https://www.mdpi.com/2073-4425/12/2/304/s1, Table S1: List of animals, Table S2: List of mRNA and protein reference sequences of ungulates, Table S3: Primers and PCR conditions used for amplification of genes, Table S4: PCR protocols, Table S5: Qualimap statistics for sequences mapped to *C. dromedarius* reference, Figure S1: Variability of perforin protein in camelids, Figure S2: Variability of granulysin protein in camelids, Figure S3: Variability of trypsin-like locus granzymes in camelids, Figure S4: Variability of chymotrypsin-like locus granzymes in camelids, Figure S5: Variability of metase-like locus granzyme in camelids, Figure S6: Comparison of mapped reads for dromedary camel *GZMB* amplicons, Figure S7: Comparison of mapped reads for Bactrian camel *GZMB* amplicons.
