# Supplementary material for: A Deadly Cargo: Gene Repertoire of Cytotoxic Effector Proteins in the Camelidae
Source: Genes (Basel). 2021 Feb 21;12(2):304. doi: 10.3390/genes12020304 (PMC7924851; doi:10.3390/genes12020304)
Supplement: Supplementary file 1 [file genes-12-00304-s001.pdf]

Table S1. List of camels studied

Lab number	Place of collection	Country of origin	Year of collection	Sample type	Sex	Age (years)
C. dromedarius 413	Jordan, Irbid	Qatar	2010	hair plucked	F	10
C. dromedarius 418	Jordan, Irbid	Qatar	2010	hair plucked	F	6
C. dromedarius 419	Jordan, Irbid	Qatar	2010	hair plucked	F	7
C. dromedarius 433	Jordan, Mafraq	Jordan	2010	FTA card	F	8
C. dromedarius 668	Iran, Ahwaz	Iran	2011	DNA extract	Un	Un
C. dromedarius 795A	Saudi Arabia, Al Jouf	Saudi Arabia	2013	FTA card	F	Un
C. dromedarius 799A	Saudi Arabia, Al Jouf	Saudi Arabia	2013	FTA card	M	Un
C. dromedarius 800A	Saudi Arabia, Al Jouf	Saudi Arabia	2013	FTA card	M	Un
C. dromedarius 801A	Austria, Eithental	Canary Islands	2013	EDTA blood	F	Un
C. dromedarius 804A	UAE, Dubai	UAE	2013	FTA card	F	Un
C. dromedarius 815A	Sudan, Karthoum	Sudan	2013	FTA card	F	8
C. dromedarius 818A	Pakistan	Pakistan	2013	FTA card	M	Un
C. dromedarius 852	Nigeria	Nigeria	2013	FTA card	Un	Un
C. dromedarius 891	Kazakstahn, west	Kazakstahn	2015	FTA card	Un	Un
C. dromedarius 893	Kazakstahn, west	Kazakstahn	2015	FTA card	Un	Un
Bactrian_35	Mongolia, Norovlin	Mongolia	2009	FTA card	M	8
Bactrian_53	Mongolia, Norovlin	Mongolia	2009	FTA card	M	7
Bactrian_56	Mongolia, Norovlin	Mongolia	2009	FTA card	M	8
Bactrian_159	Mongolia, Bayan Ovoo	Mongolia	2009	FTA card	F	7
Bactrian_176	Mongolia, Bayan Ovoo	Mongolia	2009	FTA card	M	5
Bactrian_186	Mongolia, Bayan Ovoo	Mongolia	2009	FTA card	M	10
Bactrian_191	Mongolia, Bayan Ovoo	Mongolia	2009	FTA card	M	3
Bactrian_222	Mongolia, Galshar	Mongolia	2009	FTA card	F	4
Bactrian_253	Mongolia, Galshar	Mongolia	2009	FTA card	F	8
Bactrian_259	Mongolia, Galshar	Mongolia	2009	FTA card	M	6

Un - unknown

Table S2. List of mRNA and protein reference sequences of ungulates

**locus**	**PRF1**		**GNLY**	
Camelus dromedarius	XM_031461138.1	XP_031316998.1	XM_010997841.2	XP_010996143.1
Camelus bactrianus	XM_010951709.1*	XP_010950011.1*	XM_010958936.1	XP_010957238.1
Camelus ferus	XM_006181161.3	XP_006181223.3	XM_014558318.2	XP_014413804.1
Vicugna pacos	XM_006212875.3*	XP_006212937.2*	XM_015238746.2	XP_015094232.1
Bos taurus	XM_005226334.4	XP_005226391.1	XM_024999424.1	XP_024855192.1
Bos indicus	XM_019954264.1	XP_019809823.1	XM_019970320.1	XP_019825879.1
Bos mutus	XM_005904235.2	XP_005904297.2	XM_005891665.1	XP_005891727.1
Capra hircus	XM_005699151.3	XP_005699208.1	XM_005686713.2<br>XM_005686712.3	XP_005686770.1<br>XP_005686769.1
Ovis aries	XM_004021436.4	XP_004021485.2	XM_027965892.1<br>XM_027965710.1	XP_027821693.1<br>XP_027821511.1
Sus scrofa	XM_003483492.4	XP_003483540.2	NM_001278755.1	NP_001265684.1
Equus caballus	XM_001502776.5	XP_001502826.3	NM_001081929.3	NP_001075398.2
Equus asinus	XM_014840306.1	XP_014695792.1	XM_014852808.1*	XP_014708294.1*

* - corrected

NK-lysin

granulysin

Table S2 (continued)

locus	GZMB		GZMH	
Camelus dromedarius	XM_010986180.2	XP_010984482.2	XM_031453514.1	XP_031309374.1
Camelus bactrianus	XM_010963026.1*	XP_010961328.1*	XM_010954801.1	XP_010953103.1
Camelus ferus	XM_006182831.3	XP_006182893.2	XM_032481798.1<br>XM_032481790.1<br>XM_032481793.1	XP_032337689.1<br>XP_032337681.1<br>XP_032337684.1
Vicugna pacos	XM_031678781.1*	XP_031534641.1*	XM_031678893.1	XP_031534753.1
Bos taurus	XM_002696646.5	XP_002696692.1	XM_002696688.6	XP_002696734.2
Bos indicus	XM_019983272.1	XP_019838831.1	XM_019984053.1	XP_019839612.1
Bos mutus	XM_005904884.2	XP_005904946.1	XM_005904885.2	XP_005904947.1
Capra hircus	XM_018065884.1<br>XM_018045289.1	XP_017921373.1<br>XP_017900778.1	XM_018066527.1	XP_017922016.1
Ovis aries	XM_027957506.1<br>XM_027957507.1	XP_027813307.1<br>XP_027813308.1	XM_027957214.1<br>XM_027957210.1*	XP_027813015.1<br>XP_027813011.1*
Sus scrofa	NM_001143710.1	NP_001137182.1	NM_001143693.1	NP_001137165.1
Equus caballus	NM_001081881.1<br>XM_023623277.1	NP_001075350.1<br>XP_023479045.1	XM_005613551.3<br>XM_001914736.5	XP_005613608.2<br>XP_001914771.2
Equus asinus	XM_014839985.1<br>XM_014839986.1	XP_014695471.1<br>XP_014695472.1	XM_014851359.1	XP_014706845.1

* - corrected

different annotation

Table S2 (continued)

locus	GZMA		GZMO	
Camelus dromedarius	XM_010977771.2	XP_010976073.1	XM_031440963.1	XP_031296823.1
Camelus bactrianus	XM_010966291.1	XP_010964593.1	XM_010966292.1	XP_010964594.1
Camelus ferus	XM_006185929.3	XP_006185991.1	XM_032467654.1	XP_032323545.1
Vicugna pacos	XM_006205994.3	XP_006206056.1	XM_031673562.1	XP_031529422.1
Bos taurus	NM_001099095.1	NP_001092565.1	NM_001001142.1	NP_001001142.1
Bos indicus	XM_019983132.1*	XP_019838691.1*	XM_019982727.1	XP_019838286.1
Bos mutus	XM_005887373.1	XP_005887435.1	XM_005887372.2	XP_005887434.1
Capra hircus	XM_005694709.2	XP_005694766.2	XM_013972764.2	XP_013828218.2
Ovis aries	XM_004016990.4	XP_004017039.2	XM_004016991.3	XP_004017040.2
Sus scrofa	NM_001198926.1	NP_001185855.1	NM_001143709.1	NP_001137181.1
Equus caballus	XM_001494044.5	XP_001494094.2	XR_001379369.2	
Equus asinus	XM_014860332.1	XP_014715818.1	XR_001401172.1	

* - corrected

different annotation

long non-coding RNA

Table S2 (continued)

locus	GZMK		GZMM	
Camelus dromedarius	XM_010977773.2	XP_010976075.1	XM_010985277.2	XP_010983579.1
Camelus bactrianus	XM_010966293.1	XP_010964595.1	XM_010966605.1	XP_010964907.1
Camelus ferus	XM_006185927.2	XP_006185989.1	XM_014551981.2	XP_014407467.2
Vicugna pacos	XM_006205992.3	XP_006206054.1	XM_015241228.2	XP_015096714.1
Bos taurus	NM_001205959.1	NP_001192888.1	XM_002689139.5	XP_002689185.1
Bos indicus	XM_019982730.1*	XP_019838289.1*	XM_019963642.1*	XP_019819201.1*
Bos mutus	XM_005887371.1	XP_005887433.1	XM_005892744.2	XP_005892806.1
Capra hircus	XM_013972766.2	XP_013828220.1	XM_005682880.3	XP_005682937.2
Ovis aries	XM_004016992.3	XP_004017041.2	XM_015096005.2	XP_014951491.1
Sus scrofa	NM_001143711.1	NP_001137183.1	NM_001143712.1	NP_001137184.1
Equus caballus	XM_001497014.6	XP_001497064.1	XM_023644216.1*	XP_023499984.1*
Equus asinus	XM_014860329.1	XP_014715815.1	XM_014845744.1*	XP_014701230.1*

* - corrected

Table S3. Primers and PCR conditions used for amplification of genes

Locus	Forward primer	Reverse primer	PCR protocol	Annealing temperature
PRF1	5′-GGGCAGGAAGCAGAAGTGAT-3′	5′-GCCAGAACCGTCACCATCAT-3′	A	63°C
GNLY	5′-AGCgCTGAGGTACCACTCCT-3′	5′-GTGTGAGAGCTGCTAAGGCT-3′	A	63°C
GZMB	5′-ACCCAGAGAACATCATCAGGTAGAC-3′	5′-CTGTGAATGGGGGTCAACAAAT-3′	A	63°C
GZMH	5′-CCACATCAGAGTAGCCAGAGC-3′	5′-TGCATATCAGAAGTGGTCGC-3′	A	63°C
GZMK	5′-TTGCACCTGGTACGAACAGA-3′	5′-CTGGTTGAAGGTGGTGCCATA-3′	B	63°C
GZMO	5′-CCTTTGTCATTTCTGCCTGTGT-3′	5′-GGCTGGACTGAGGCTATTGA-3′	B	58°C
GZMA	5′-TGCCCTTGGCCAATTACTCC-3′	5′-TCCCAGCCTGACCTTAGTGT-3′	B	63°C
GZMM	5′-GTGAGCTCATCCACTACGGG-3′	5′-TCGGTTGTCCTTACGTCGC-3′	A	63°C

Table S4. PCR protocols

**PCR protocol A**		
5x KAPA A buffer	2.5 µl	thermocycler program:
5x KAPA Enhancer	2.5 µl	95°C 3 min
10mM dNTPs (each)	0.25 µl	35 rounds of [95°C 25 sec; annealing 15 sec; 72°C 30 sec per kb]
10µM forward prime	0.625 µl	72°C 1 min per kb
10µM reverse prime	0.625 µl	hold at 8°C
KAPA 2G HotStart Polymerase 5U/µl	0.1 µl	
H_2O	add to 12.5 µl	
genomic DNA	50 ng	
**PCR protocol B**		
5x Expand LR buffer	2.5 µl	thermocycler program:
10mM dNTPs (each)	0.625 µl	92°C 2 min
10µM forward prime	0.5 µl	10 rounds of [92°C 10 sec; annealing 20 sec; 68°C 1 min per kb]
10µM reverse prime	0.5 µl	25 rounds of [92°C 10 sec; annealing 15 sec; 68°C 1 min per kb+20 sec per cycle]
Expand Long Range Enzyme 5U/µl	0.175 µl	68°C 7 min
H_2O	add to 12.5 µl	hold at 8°C
genomic DNA	100 ng	

Table S5. Qualimap statistics for sequences mapped to *C. dromedarius* reference

PRF1 4635 bp	Animal	GenBank accession	Mapped bases	Mean coverage	Standard deviation	GNLY 4341 bp	Animal	GenBank accession	Mapped bases	Mean coverage	Standard deviation
C. dromedarius	413	MW456757	2584095	557.5178	249.6592	*C. dromedarius*	413	MW456777	4009068	923.5356	680.1411
	418	MW456758	6191623	1,335.8410	569.8394		418	MW456778	7259736	1,672.3649	1,213.7646
	795A	MW456759	3863736	833.2405	290.1930		795A	MW456779	1167511	268.9498	209.2209
	799A	MW456760	2246415	484.6634	214.3791		799A	MW456780	3959732	912.1705	644.4853
	800A	MW456761	4140408	893.2919	372.9841		800A	MW456781	2422012	557.9387	415.4826
	801A	MW456762	12749625	2,750.7282	1,004.1147		801A	MW456782	7859618	1,810.5547	1,177.7831
	804A	MW456763	3392539	731.9394	308.5080		804A	MW456783	4629706	1,066.5068	764.3690
	818A	MW456764	1647998	355.5551	146.1169		818A	MW456784	4231683	974.8176	694.9904
	852	MW456765	5055745	1,090.7756	434.5966		852	MW456785	8464552	1,949.9083	1,416.3976
	893	MW456766	1220075	263.2309	115.4031		893	MW456786	9178789	2,114.4411	1,370.1051
C. bactrianus	35	MW456767	4923083	1,062.1538	417.5495	*C. bactrianus*	35	MW456787	9727274	2,240.7911	1,397.3192
	53	MW456768	183774	39.6492	28.1296		53	MW456788	6491793	1,495.4603	890.5096
	56	MW456769	5685063	1,226.5508	514.1138		56	MW456789	2644360	609.1592	434.6159
	159	MW456770	6717441	1,449.2861	612.4809		159	MW456790	5324314	1,226.5179	843.4950
	176	MW456771	9632489	2,078.2069	837.2529		176	MW456791	3923765	903.8850	663.7177
	186	MW456772	4945840	1,067.0636	439.7716		186	MW456792	3102854	714.7786	490.8996
	191	MW456773	3600864	776.8854	332.3984		191	MW456793	7069785	1,628.6075	1,054.7014
	222	MW456774	3027276	653.1340	294.5174		222	MW456794	1047058	241.2020	176.7368
	253	MW456775	12866625	2,775.9709	1,084.4135		253	MW456795	4380500	1,009.0993	676.8732
	259	MW456776	15272904	3,295.1249	1,304.2172		259	MW456796	4220107	972.1509	862.4902

Table S5. (continued)

GZMK 9411 bp	Animal	GenBank accession	Mapped bases	Mean coverage	Standard deviation
C. dromedarius	418	MW456797	5990975	636.5928	196.4658
	419	MW456798	3883813	412.6887	423.8372
	433	MW456799	8584624	912.1904	285.0234
	795A	MW456800	1535463	163.1562	51.3064
	799A	MW456801	25438525	2,703.0629	1,567.4700
	800A	MW456802	8305378	882.5181	233.6026
	801A	MW456803	16350123	1,737.3417	503.6328
	804A	MW456804	12301761	1,307.1683	461.8240
	852	MW456805	15784311	1,677.2193	549.5827
	893	MW456806	8502558	903.4702	259.1690
C. bactrianus	35	MW456807	8079258	858.4909	270.9955
	53	MW456808	3757483	399.2650	110.4478
	56	MW456809	8585706	912.3054	241.6669
	159	MW456810	18033123	1,916.1750	596.7837
	176	MW456811	16755990	1,780.4686	430.6197
	186	MW456812	8467543	899.7495	232.5611
	191	MW456813	24364025	2,588.8880	910.5357
	222	MW456814	3816127	405.4964	132.7636
	253	MW456815	26315083	2,796.2048	833.5345
	259	MW456816	28221978	2,998.8288	1,444.7609

GZMA 7578 bp	Animal	GenBank accession	Mapped bases	Mean coverage	Standard deviation
C. dromedarius	419	MW456817	3645050	481.0042	189.8943
	433	MW456818	4168709	550.1068	199.6063
	795A	MW456819	2586852	341.3634	131.5388
	799A	MW456820	23990865	3,165.8571	962.9101
	800A	MW456821	10236008	1,350.7532	436.4234
	801A	MW456822	21853137	2,883.7605	922.1726
	804A	MW456823	7139846	942.1808	305.3025
	818A	MW456824	19094078	2,519.6725	870.2883
	852	MW456825	20922191	2,760.9120	865.3538
	893	MW456826	17327683	2,286.5773	734.3595
C. bactrianus	35	MW456827	22823852	3,011.8570	916.6027
	53	MW456828	15019782	1,982.0245	563.3386
	56	MW456829	22203843	2,930.0400	961.7492
	159	MW456830	16713427	2,205.5195	741.9538
	176	MW456831	20156843	2,659.9159	920.1382
	186	MW456832	12980131	1,712.8703	530.7982
	191	MW456833	31262782	4,125.4661	1,220.6118
	222	MW456834	9863703	1,301.6235	402.7387
	253	MW456835	31519563	4,159.3511	1,274.4402
	259	MW456836	8983904	1,185.5244	422.4730

Table S5. (continued)

GZMO 10183 bp	Animal	GenBank accession	Mapped bases	Mean coverage	Standard deviation	GZMM 5913 bp	Animal	GenBank accession	Mapped bases	Mean coverage	Standard deviation
C. dromedarius	419	MW456837	5091991	500.0482	259.7189	*C. dromedarius*	413	MW456897	7623183	1,289.2243	829.6640
	433	MW456838	5341874	524.5875	296.2394		418	MW456898	3145807	532.0154	352.3627
	795A	MW456839	8618541	846.3656	446.4670		795A	MW456899	250712	42.4001	26.2102
	799A	MW456840	10014986	983.5005	349.2530		799A	MW456900	6312504	1,067.5637	681.0508
	800A	MW456841	8696345	854.0062	459.4756		800A	MW456901	1624481	274.7304	184.0273
	801A	MW456842	9214852	904.9251	372.4556		801A	MW456902	4410260	745.8583	481.3960
	804A	MW456843	13847477	1,359.8622	569.3312		804A	MW456903	4744852	802.4441	505.8241
	818A	MW456844	5958895	585.1807	205.8962		818A	MW456904	3254000	550.3129	379.4431
	852	MW456845	5178426	508.5364	296.0241		852	MW456905	2668851	451.3531	318.7493
	893	MW456846	21987736	2,159.2592	782.6109		893	MW456906	1514299	256.0966	170.7201
C. bactrianus	35	MW456847	3816313	374.7730	122.8859	*C. bactrianus*	35	MW456907	3576151	604.7947	436.5031
	53	MW456848	2524827	247.9453	152.4585		53	MW456908	2254020	381.1974	274.4737
	56	MW456849	4333215	425.5342	247.9790		56	MW456909	1600337	270.6472	193.8532
	159	MW456850	4454761	437.4704	138.6560		159	MW456910	2423611	409.8784	295.6068
	176	MW456851	7884833	774.3133	406.7455		176	MW456911	2854338	482.7225	342.1910
	186	MW456852	8208219	806.0708	248.6689		186	MW456912	1339799	226.5853	167.8066
	191	MW456853	15152571	1,488.0262	393.1216		191	MW456913	3385021	572.4710	409.9238
	222	MW456854	7230036	710.0104	232.8207		222	MW456914	829225	140.2376	98.7235
	253	MW456855	26437549	2,596.2436	819.9566		253	MW456915	13062721	2,209.1529	1,456.2110
	259	MW456856	33034987	3,244.1311	1,691.7262		259	MW456916	9345633	1,580.5231	1,161.1126

Table S5. (continued)

GZMB 3414 bp	Animal	GenBank accession	Mapped bases	Mean coverage	Standard deviation	GZMH 5552 bp	Animal	GenBank accession	Mapped bases	Mean coverage	Standard deviation
C. dromedarius	418	MW456857	9950931	2,914.7425	1,349.6566	*C. dromedarius*	418	MW456877	4585341	825.8899	1,036.3396
	668	MW456858	8138573	2,383.8820	1,021.9978		668	MW456878	2008063	361.6828	509.2808
	795A	MW456859	8079950	2,366.7106	960.6279		795A	MW456879	1447429	260.7041	111.1113
	799A	MW456860	2061496	603.8360	311.7032		799A	MW456880	2902364	522.7601	239.7535
	800A	MW456861	8950045	2,621.5715	1,079.5493		800A	MW456881	2648579	477.0495	201.8859
	801A	MW456862	8163530	2,391.1921	1,022.4830		801A	MW456882	2964132	533.8854	1,103.0225
	804A	MW456863	8764385	2,567.1895	1,058.7604		804A	MW456883	2721283	490.1446	777.9230
	815A	MW456864	13940945	4,083.4637	1,783.8839		815A	MW456884	2653495	477.9350	847.2787
	852	MW456865	7243987	2,121.8474	1,010.4887		852	MW456885	2199035	396.0798	170.8361
	891	MW456866	3588896	1,051.2291	479.5665		893	MW456886	3239725	583.5240	243.7391
C. bactrianus	35	MW456867	10426567	3,054.0618	1,279.1850	*C. bactrianus*	35	MW456887	2700771	486.4501	813.7304
	53	MW456868	12187897	3,569.9757	1,655.9338		53	MW456888	1995915	359.4948	609.8218
	56	MW456869	5861947	1,717.0319	812.0062		56	MW456889	1179644	212.4719	404.9877
	159	MW456870	8897359	2,606.1391	1,125.7722		159	MW456890	1089571	196.2484	355.0098
	176	MW456871	9140072	2,677.2326	1,162.1572		176	MW456891	1883924	339.3235	610.8709
	186	MW456872	7089251	2,076.5234	936.7129		186	MW456892	2241289	403.6904	732.7212
	191	MW456873	10031196	2,938.2531	1,266.3133		191	MW456893	2445616	440.4928	199.6274
	222	MW456874	1994217	584.1292	282.4676		222	MW456894	2976668	536.1434	229.8691
	253	MW456875	7168334	2,099.6878	1,087.1732		253	MW456895	2341318	421.7071	190.3380
	259	MW456876	9885890	2,895.6913	1,353.9999		259	MW456896	4703811	847.2282	1,433.7298

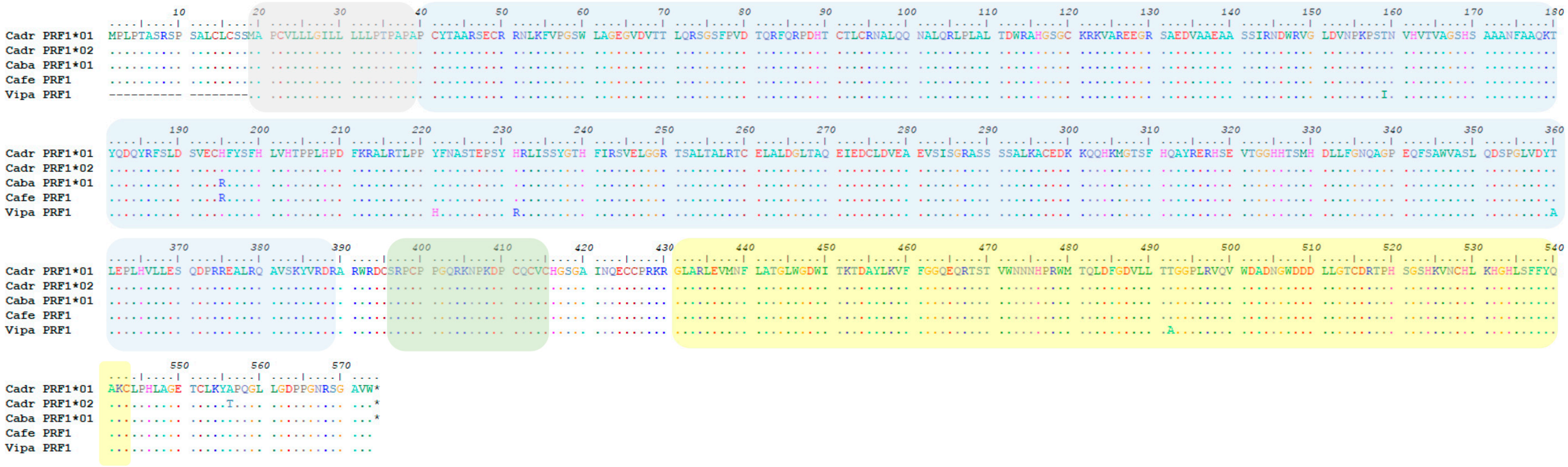

Figure S1. Variability of perforin protein in camelids. The alignment of *PRF1* amino acid sequences identified in a panel of dromedary (*Cadr*) and Bactrian camels (*Caba*) with reference sequences of wild camel (*Cafe*) and alpaca (*Vipa*). Organization of protein domains is depicted by color rectangles according to human perforin (NP_005032.2): *grey* – signal peptide; *blue* – pore-forming membrane attack complex and perforin (MACPF) domain with two membrane-penetrating regions (positions 130-183 and 258-314, position 232 is important for oligomerization); *green* – epidermal grow factor-like domain; *yellow* – calcium (positions 448,454,502,504 and 510) and membrane binding C2 domain; positions 223 and 567 – N-glycosylation sites. *Dot* – identical amino acid residue; *dash* – gap in alignment; *asterisk* – stop codon.

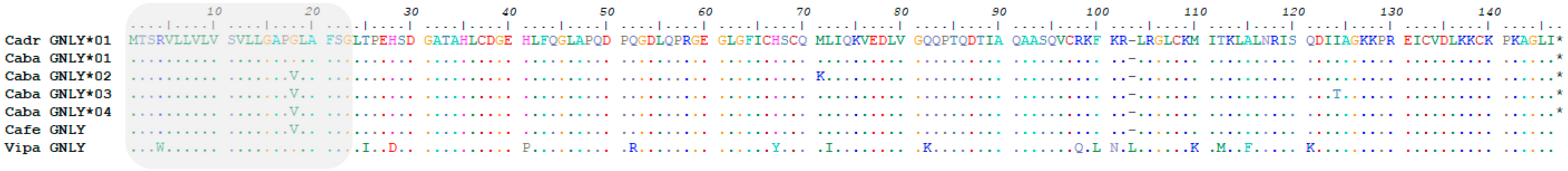

Figure S2. Variability of granulysin protein in camelids. The alignment of *GNLY* amino acid sequences identified in a panel of dromedary (*Cadr*) and Bactrian camels (*Caba*) with reference sequences of wild camel (*Cafe*) and alpaca (*Vipa*). *Grey rectangle* – signal peptide; *dot* – identical amino acid residue; *dash* – gap in alignment; *asterisk* – stop codon.

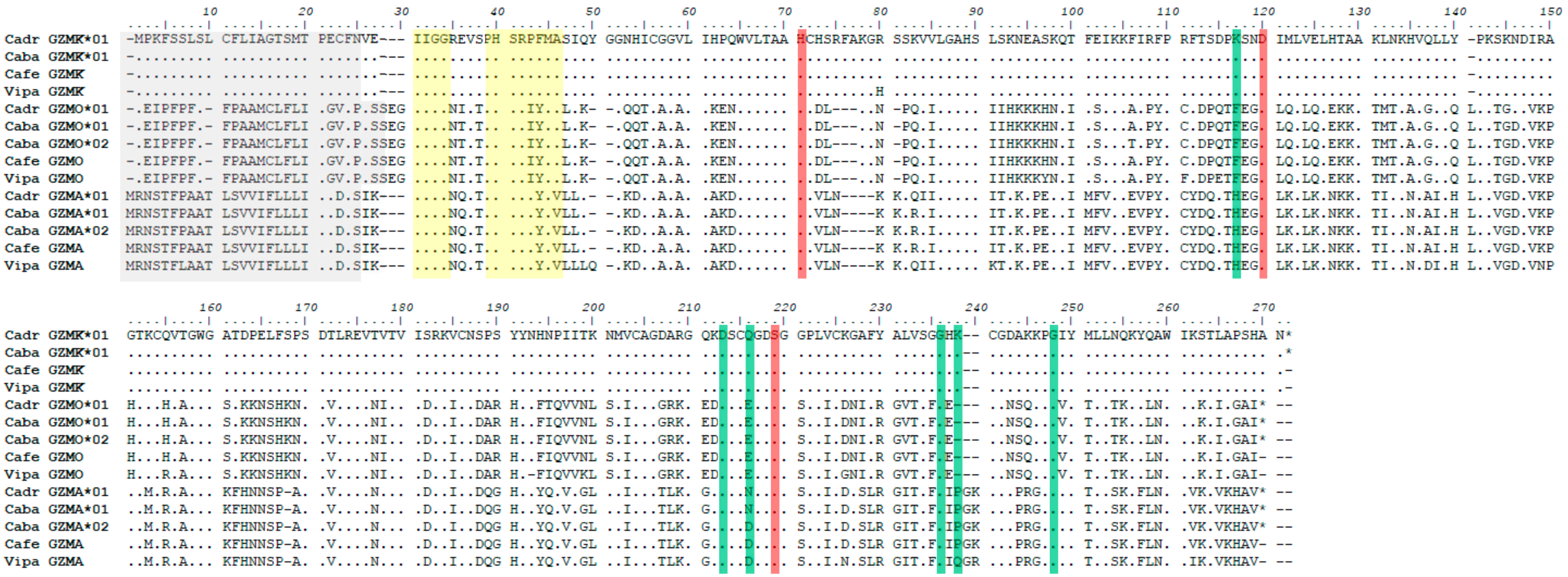

Figure S3. Variability of trypsin-like locus granzymes in camelids. The amino acid sequences of granzyme K (*GZMK*), granzyme O (*GZMO*) and granzyme A (*GZMA*) identified in a panel of dromedary (*Cadr*) and Bactrian camels (*Caba*) were aligned with reference sequences of wild camel (*Cafe*) and alpaca (*Vipa*). Highlighted are conserved consensus sequences: *grey* – signal peptide; *yellow* – N-terminal positions 1-4 (IIGG) and 9-16 (PHSRPYMA) of mature enzyme; *red* – amino acids of catalytic triad; *green* – substrate-determining residues (S1- positions 213, 236, 248 and 216; S2 – 117; S3 – 238). Conserved cysteine residues that form disulfide bonds are at positions: 56, 72, 154, 186, 204, 215, 225 and 241 of the alignment. *Dot* – identical amino acid residue; *dash* – gap in alignment; *asterisk* – stop codon.

```
                           10         20         30         40         50         60         70         80         90        100        110        120        130        140        150
               ....|....| ....|....| ....|....| ....|....| ....|....| ....|....| ....|....| ....|....| ....|....| ....|....| ....|....| ....|....| ....|....| ....|....| ....|....|
Cadr GZMB*01   MQPL--LLLL PLAFLLPPGT NAGEIIGGHE AKPHSRPYMA YLQIWNQDVR SRCGGFLIRE DFVLTAAHCW GSSINVTLGA HNIKKQERTQ QVIPVRKAIR HPDYNKRNYA NDIMLLQLQR KAKQTAAVRP LRLPGDRARV KPGQACDVAG
Cadr GZMB*02   ....LL.... .......... .......... .......... .......... .......... .......... .......... .......... .......... .......... .......... .......... .......... ..........
Cadr GZMB*03   ....LL.... .......... .......... .......... .......... .......... .......... .......... .......... .......... .......... .......... .......... .......... ..........
Caba GZMB*01   ....LL.... .......... .......... .......... ........D. .......... .......... .......... .......... .......... .......... .......... .......... .....G.... ..........
Caba GZMB*02   ....LL.... .......... .......... .......... ........D. .......... .......... .......... .......... .......... .......... .......... .......... .....G.... ..........
Caba GZMB*03   ....LL.... .......... .......... .......... ........D. .......... .......... .......... ........N. .......... .......... .......... .......... .....G.... ..........
Caba GZMB*04   ....LL.... .......... .......... .......... ........D. .....V.... .......... .......... ........N. .......... .......... .......... .......... .....G.... ..........
Caba GZMB*05   ....LL.... .......... .......... .......... ........DQ .......... .......... .......... .......... .......... .......... .......... .......... .....G.... ..........
Cafe GZMB      ....LL.... .......... .......... .......... ........D. .......... .......... .......... .......... .......... .......... .......... .......... .....G.... ..........
Vipa GZMB      ....--.... .......... .......... .......... ........D. .......... .......... .......... .......... .......... .......... ........E. ...R...... .GC..G.... ..........
Cadr GZMH*01   ....LLVMAV L.PAGRGQPS LS........ .......... FV.FLD.ERM R....V.VQK .........R .......... .......G.. ..V...R..P .....PKDHS S......... .......... .......... ......A...
Cadr GZMH*02   ....LLVMAV L.PAGRGQPS LS........ .......... FV.FLD.ERM R....V.VQK .........R .......... .......G.. ..V...R..P .....PKDHS S......... .......... .....G.... ......A...
Caba GZMH*01   ....LLVMAV L.PAGRGQPS LS........ .......... FV.FLD.ERM R....V.VQK .........R .......... .......G.. ..V...R..P .....PKDHS S......... .......... .....G.... ......G...
Caba GZMH*02   ....LLVMAV L.PAGRGQPS LS........ .......... FV.FLD.ERM R....V.VQK .........R .......... .......G.. ..V...R..P .....PKDHS S......... .......... .......... ......G...
Caba GZMH*03   ....LLVMAV L.PAGRGQPS LS........ .......... FV.FLD.ERM R....V.VQK .........R .......... .......G.. ..V...R..P .....PKDHS S......... .......... .......... ......G...
Caba GZMH*04   ....LLVMAV L.PAGRGQPS LS........ .......... FV.FLD.ERM R....V.VQK .........R .......... .......G.. ..V...R..P .....PKDHS S......... .......... .......... ......A...
Cafe GZMH1     ....LLVMAV L.PAGRGQPS LS........ .......... FV.FLD.ERM R....V.VQK .........R .......... .......G.. ..V...R..P .....PKDHS S......... .......... .......... ......G...
Cafe GZMH2     ....LLVMAV L.PAGRGQPS LS........ .......... FV.FLD.ERM R....V.VQK .........R .......... .......G.. ..V...R..P .....PKDHS S......... .......... .......... ......G...
Cafe GZMH3     ....LLVMAV L.PAGRGQPS LS........ .......... FV.FLD.ERM R....V.VQK .........R .......... .......G.. ..V...R..P .....PKDHS S......... .......... .......... ......G...
Vipa GZMH      ....LLVMAV L.PAGRGQPS LS........ .....H.... FV.FLD.EKM R....V.VQK .........R .......... .......... ..V...R..P .....PKDHS S......... .......... .....G.... ......G...

                          160        170        180        190        200        210        220        230        240        250        260
               ....|....| ....|....| ....|....| ....|....| ....|....| ....|....| ....|....| ....|....| ....|....| ....|....| ....|....| ....
Cadr GZMB*01   WGRVAVAMNN YPDTLQEVKL IVQEDQKCES HLRNYYNNII QLCVGDPKKK KASFKGDSGG PLVCDNVAQG IVSYGQNDGS TPRACTKVSS FLPWIKKTMK SLQLQEPGRL PWS*
Cadr GZMB*02   .......... .......... .......... .......... .......... .......... .......... .......... .......... .......... .......... ...*
Cadr GZMB*03   .......... .......... .......... .......... .......... .......... .......... .......... .......... .......... ........H. ...*
Caba GZMB*01   .......... .......... .......... .......... .......... .......... .......... ......K... .......... .......... ........H. ...*
Caba GZMB*02   ..Q....KD. .......... .......... .......... .......... .......... .......... .......... ..Q....... .......... ........H. ...*
Caba GZMB*03   .......... .......... .......... .......... .......... .......... .......... ......K... .......... .......... ........H. ...*
Caba GZMB*04   .......... .......... .......... .......... .......... .......... .......... ......K... .......... .......... ........H. ...*
Caba GZMB*05   .......... .......... .......... .......... .......... .......... .......... ......K... .......... .......... ........H. ...*
Cafe GZMB      .......... .......... .......... .......... .......... .......... .......... ......K... .......... .......... ........H. ...-
Vipa GZMB      ..Q.V...D. .......... .......... .......K.. .......... .......... .......... ......K... .......... .......... ..H....D.. ...-
Cadr GZMH*01   ..Q...GV-- PAT....AV. T....RV... LFPG..SHAT .I.....STV .T........ ....K.LV.. .F.C.KQN.T P.GVF....H ......R... R.*------- ----
Cadr GZMH*02   ..Q...GV-- PAT....AV. T....RV... LFPG..SHAT .I.....STV .T........ ....K.LV.. .F.C.KQN.T P.GVF....H ......R... R.*------- ----
Caba GZMH*01   ..Q...GV-- PAT....AV. T....RV... LFPG..SRAT .I.....STV .T........ ....K.LV.. .F.C.KQN.T P.GVF....H ......R... R.*------- ----
Caba GZMH*02   ..Q...GV-- PAT....AV. T....RV... LFPG..SRAT .I.....STV .T........ ....K.LV.. .F.C.KQN.T P.GVF....H ......R... R.*------- ----
Caba GZMH*03   ..Q...GV-- PAT....AV. T....HV... LFPG..SRAT .I.....STV .T........ ....K.LV.. .F.C.KQN.T P.GVF....H ......R... R.*------- ----
Caba GZMH*04   ..Q...GV-- PAT....AV. T....RV... LFPG..SHAT .I.....STV .T........ ....K.LV.. .F.C.KQN.T P.GVF....H ......R... R.*------- ----
Cafe GZMH1     ..Q...GV-- PAT....AV. T....RV... LFPG..SRAT .I.....STV .T........ ....K.LV.. .F.C.KQN.T P.GVF....H ......R... R.-------- ----
Cafe GZMH2     ..Q...GV-- PAT....AV. T....RV... LFPG..SRAT .I.....STV .T........ ....K.LV.. .F.C.KQN.T P.GVF....H ......R... R.-------- ----
Cafe GZMH3     ..Q...GV-- PAT....AV. T....RV... LFPG..SRAT .I.....STV .T........ ....K.LV.. .F.C.KQN.T P.GVF....H ......R... R.-------- ----
Vipa GZMH      ..Q...GV-- PAT....AV. T....RV... LFPG..SRAT .I.A...STV .T........ ....K.LV.. .F.C.KQN.K P.GVF....H ......R... R.-------- ----
```

Figure S4. Variability of chymotrypsin-like locus granzymes in camelids. The amino acid sequences of granzyme B (*GZMB*) and granzyme H (*GZMH*) identified in a panel of dromedary (*Cadr*) and Bactrian camels (*Caba*) were aligned with reference sequences of wild camel (*Cafe*) and alpaca (*Vipa*). Highlighted are conserved consensus sequences: *grey* – signal peptide; *yellow* – N-terminal positions 1-4 (IIGG) and 9-16 (PHSRPYMA) of mature enzyme; *red* – amino acids of catalytic triad; *green* – substrate-determining residues (S1- positions 202, 225, 233 and 205; S2 – 109; S3 – 227). Conserved cysteine residues that form disulfide bonds are at positions: 53, 69, 146, 178, 193 and 214 of the alignment. *Dot* – identical amino acid residue; *dash* – gap in alignment; *asterisk* – stop codon.

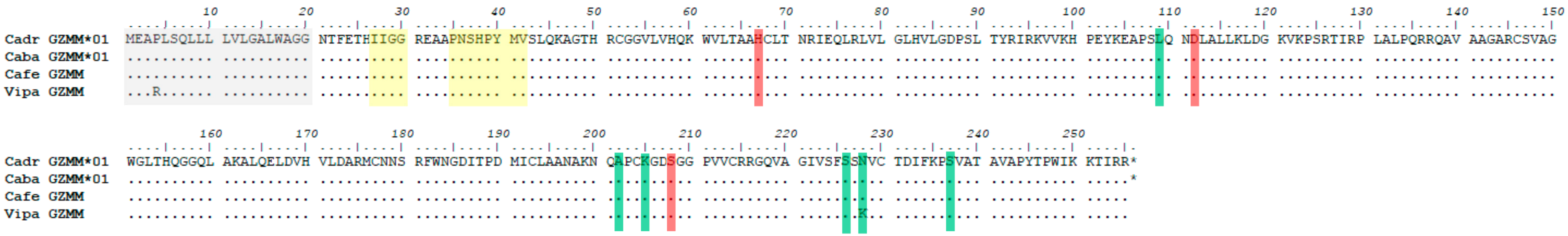

Figure S5. Variability of metase-like locus granzyme in camelids. The amino acid sequences of granzyme M (*GZMM*) identified in a panel of dromedary (*Cadr*) and Bactrian camels (*Caba*) were aligned with reference sequences of wild camel (*Cafe*) and alpaca (*Vipa*). Highlighted are conserved consensus sequences: *grey* – signal peptide; *yellow* – N-terminal positions 1-4 (IIGG) and 9-16 (PHSRPYMA) of mature enzyme; *red* – amino acids of catalytic triad; *green* – substrate-determining residues (S1- positions 202, 226, 237 and 205; S2 – 109; S3 – 228). Conserved cysteine residues that form disulfide bonds are at positions: 52, 68, 146, 177, 193, 204, 214 and 230 of the alignment. *Dot* – identical amino acid residue; *asterisk* – stop codon.

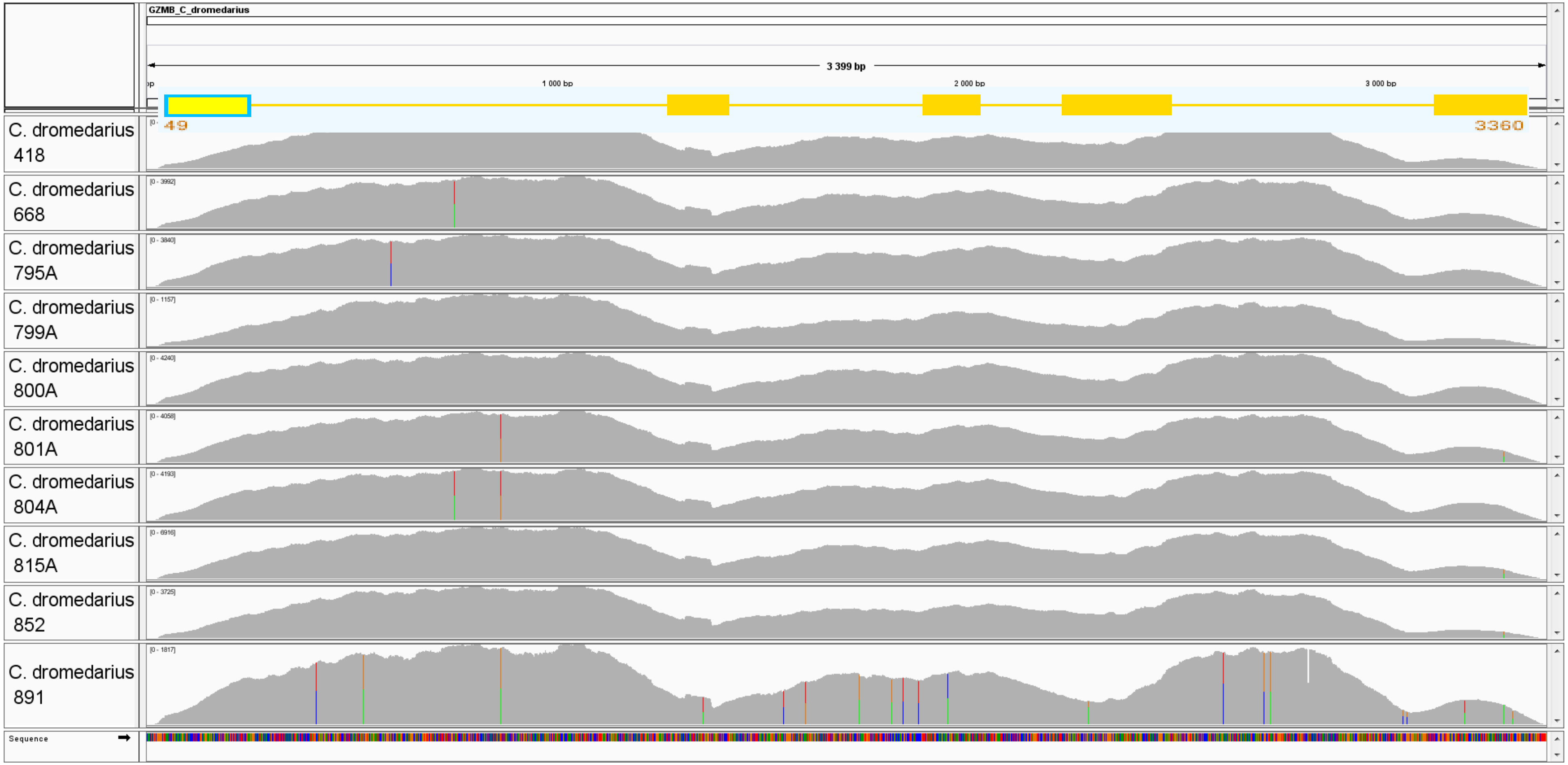

Figure S6. Comparison of mapped reads for dromedary camel *GZMB* amplicons. Each of ten tracks in IGV [33] output contains specifically indexed reads and represents individual animal genotype. Numbers in square brackets denote minimal and maximal read counts. Positions conforming to the *GZMB* reference sequence (bottom line) from *C. dromedarius* genome are in grey, variable positions are highlighted in color. Golden rectangles in the upper panel indicate locations of *GZMB* gene exons according to the mRNA reference (XM_010986180), the first exon containing 5'-untranslated region is highlighted. Camel # 891 is a heterozygote for a *C. bactrianus* haplotype.

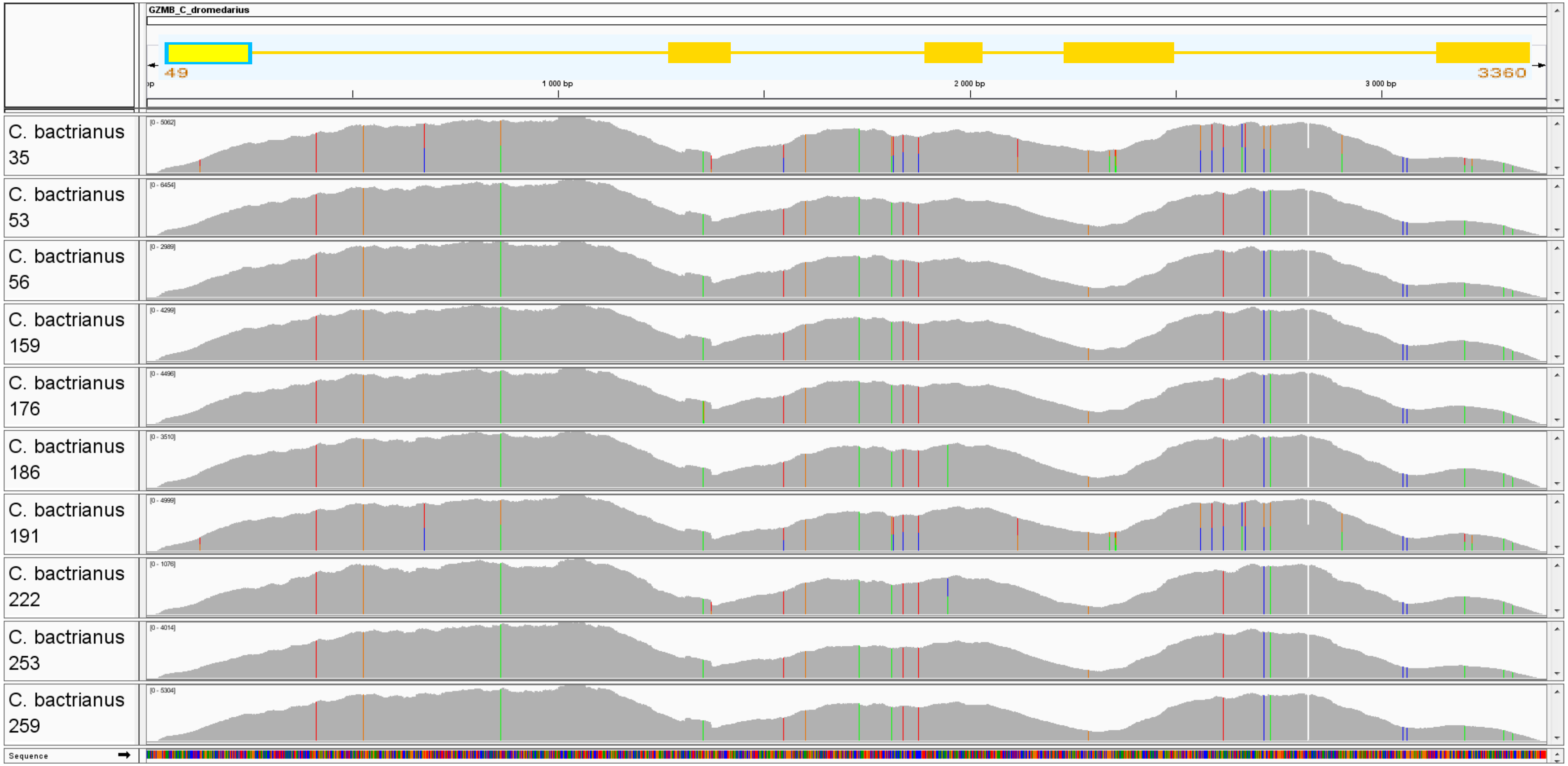

Figure S7. Comparison of mapped reads for Bactrian camel *GZMB* amplicons. Each of ten tracks in IGV [33] output contains specifically indexed reads and represents individual animal genotype. Numbers in square brackets denote minimal and maximal read counts. Positions conforming to the *GZMB* reference sequence (bottom line) from *C. dromedarius* genome are in grey, variable positions are highlighted in color. Golden rectangles in the upper panel indicate locations of *GZMB* gene exons according to the mRNA reference (XM_010986180), the first exon containing 5'-untranslated region is highlighted.
